# What makes a thriver? Unifying the concepts of posttraumatic and postecstatic growth

**DOI:** 10.3389/fpsyg.2015.00813

**Published:** 2015-06-23

**Authors:** Judith Mangelsdorf, Michael Eid

**Affiliations:** ^1^Department of Psychology, Free University of BerlinBerlin, Germany; ^2^Max Planck Institute for Human DevelopmentBerlin, Germany

**Keywords:** thriver, thriver model, posttraumatic growth, post-traumatic growth, postecstatic growth, life event, meaning making, social readjustment rating scale (SRRS)

## Abstract

The *thriver model* is a novel framework that unifies the concepts of posttraumatic and postecstatic growth. According to the model, it is not the quality of an event, but the way it is processed, that is critical for the occurrence of post-event growth. The model proposes that meaning making, supportive relationships, and positive emotions facilitate growth processes after positive as well as traumatic experiences. The tenability of these propositions was investigated in two dissimilar cultures. In Study 1, participants from the USA (*n* = 555) and India (*n* = 599) answered an extended version of the Social Readjustment Rating Scale to rank the socioemotional impact of events. Results indicate that negative events are perceived as more impactful than positive ones in the USA, whereas the reverse is true in India. In Study 2, participants from the USA (*n* = 342) and India (*n* = 341) answered questions about the thriver model's main components. Results showed that posttraumatic and postecstatic growth are highly interrelated. All elements of the thriver model were key variables for the prediction of growth. Supportive relationships and positive emotions had a direct effect on growth, while meaning making mediated the direct effect of major life events.

## Introduction

A survivor is a person who lived through hardship or disaster. A *thriver* is more than that. It is someone who not only goes through an exceptionally positive or threatening life event, but shows subsequent growth because of the experience. Why do some people thrive after life's worst and best experiences while others stay the same?

A large number of studies had aimed to find an answer to this question. In the last decades, an increasing body of research has suggested that highly stressful experiences are a possible facilitator of personal change processes (e.g., Park et al., 1996; Tedeschi and Calhoun, 1996; Joseph and Linley, 2004). Tedeschi and Calhoun (1996) established the notion of posttraumatic growth (PTG) for this phenomenon, which has also been referred to as *stress-related growth* (Park et al., 1996; Moore et al., 2010; LoSavio et al., 2011), *adversarial growth* (Joseph and Linley, 2004; Fortune et al., 2005), and *benefit-finding* (Affleck and Tennen, 1996).

Consistent with the work of Tedeschi and Calhoun (1996, 2004), most research on growth after major life events has focused exclusively on negative experiences as possible catalysts for positive development (e.g., Joseph and Linley, 2004; Swickert and Hittner, 2009; Larner and Blow, 2011). This uni-directional approach has led to the perception that primarily negative events can result in accelerated complex positive change processes, which are referred to here as growth. An example is the early research on life themes (Csikszentmihalyi and Beattie, 1979). Csikszentmihalyi and Beattie (1979) suggest that by undergoing problematic life events in childhood, individuals might develop life themes that are critical for their later life path, life story, and interpretation of reality. The authors found evidence for the existence of life themes and their impact on one's later life. Simultaneously, since their basic assumption was that life themes are always based on problematic events, they only included cases that met the criterion that a problematic experience was identified. Hereby, they excluded the possibility that positive life events might result in life themes as well. Supporting this assumption, Baumeister et al. (2001) argue that there is no corresponding positive concept to trauma, and infer the greater impact and importance of negative events. They conclude: “Bad is stronger than good” (Baumeister et al., 2001, p. 323).

While several research reviews support the assumption that bad is stronger than good (e.g., Baumeister et al., 2001; Rozin and Royzman, 2001; Eby et al., 2010), there are critical limitations to them. Most of the research on the predominant role of negative experiences is limited by one critical factor; they are based on Western, mostly American populations. This fact leaves a key question unanswered: Is the negativity bias a cultural artifact?

Studies on post-event growth have mainly evaluated personal development as a function of the negative stress level of a challenging event (e.g., Kesimci et al., 2005; Kashdan and Kane, 2011). They found evidence for a positive relationship between experienced posttraumatic distress and posttraumatic growth (Frazier et al., 2001), while other studies found a negative relation (Park et al., 1996). Meanwhile, based on the assumption that a negative disruption of core beliefs enables growth (Cann et al., 2010), most of these studies excluded events that are perceived as positive at the time they happen.

In the last years, a new perspective on human flourishing is found in research examining the possibility of growth after emotional peak experiences (Keltner and Haidt, 2003; Taubman-Ben-Ari et al., 2012; Roepke, 2013). Different authors argue that life events which enhance positive emotions, such as awe and elevation, can also foster personal development (Keltner and Haidt, 2003; Fredrickson, 2004; Taubman-Ben-Ari et al., 2012). In her pioneering research, Roepke (2013) termed this phenomenon *postecstatic growth* (PEG). Concepts, such as the *broaden-and-build theory* (Fredrickson, 2004) and the *inspire-and-rewire hypothesis* (Keltner and Haidt, 2003) provide theoretical frameworks for the idea of thriving after highly positive experiences, including moral growth and a deepening of close relationships. In support of this hypothesis, Berntsen et al. (2011) conducted a study with over 2000 adults rating the Centrality of Event Scale (CES) as well as different measures of well-being, posttraumatic stress disorder, and depression. Participants reported, that with the passage of time, the centrality of negative events decreased, while their positive life events became more central to them. They found that highly positive life events are considerably more central to an individual's identity and personal life story than negative ones. While the importance of positive experiences increases over time, the one of negative experiences diminishes (Berntsen et al., 2011). This assumption is also supported by earlier studies on the relation of life scripts, autobiographical memory, and major life events (e.g., Rubin and Berntsen, 2003). Thus, one could argue that, in the long run: Good is stronger than bad. However, from a growth perspective, does it really make a difference if a person has been subjected to trauma or peak experiences?

Research on experimental disclosure of major life events showed that the positive effects of disclosure occur independent of an event's valence (Frattaroli, 2006). In a broad meta-analysis on experimental disclosure, Frattaroli showed that the effect of writing about major life events was not moderated by the valence of the event encountered. Writing about positive as well as negative events resulted in higher levels of psychological health.

Individuals who lived through posttraumatic growth typically report positive changes in the areas of relationships, spirituality, appreciation of life, openness for new possibilities, and personal strengths (Tedeschi and Calhoun, 1996; Joseph and Linley, 2004; Park and Helgeson, 2006). After emotional peak experiences people tend to report improved relationships, more meaning in life, enhanced spirituality, and more self-esteem (Roepke, 2013). Surprisingly, there is a significant overlap in the perceived benefits of both kinds of experiences, despite the obvious differences between highly positive and negative life events (see Tedeschi and Calhoun, 2004; Roepke, 2013). These similarities suggest a new perspective to examine human thriving. It is possible that PTG and PEG are cognate processes, which can be facilitated by the same factors independent of an event's emotional valence. Possibly, it is not the quality of an event, but within-person factors that influence the processes following major life events and enable personal growth.

### The thriver model

**Figure 1:**
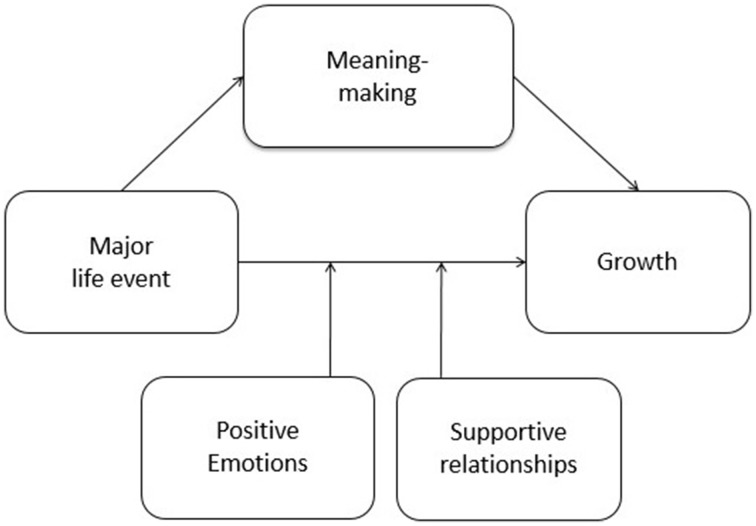
**Thriver model of contributing factors to positive development after major life events**.

The thriver model has been developed to unify psychological factors contributing to posttraumatic as well as postecstatic growth. The model is based on the assumption that people who are more likely to experience posttraumatic growth are also more likely to experience postecstatic growth and vice versa. Various research on positive changes after critical life events suggests that there are different variables that influence the occurrence of growth, such as openness (Shakespeare-Finch et al., 2003; Kashdan and Kane, 2011), severity of the stressor (Park and Helgeson, 2006), or level of traumatization (Moore et al., 2010). At the same time, there are only a few critical variables mentioned in the existing research that apply to positive and negative life events and are influenceable by the individual. The thriver model combines three well-investigated key factors that have been extracted from posttraumatic and postecstatic growth theories, facilitating positive development after major life events. The three contributing factors of the model are positive emotions (Fredrickson, 2004; Norlander et al., 2005), supportive relationships (Prati and Pietrantoni, 2009; Schroevers et al., 2010), and meaning making (Kray et al., 2010; Wong et al., 2011; Park and George, 2013). Figure 1 depicts the thriver model.

The thriver model is a process model that aims to describe how growth after major life events can be facilitated. One consequence of critical life experiences is a process referred to as core belief disruption (Cann et al., 2010). Major life events can question our general assumption of the world and hereby make it necessary to integrate the new experience into existing mental structures. The thriver model suggests that meaning making is a key process to enable integration and mental reorganization for positive and negative life events. Supportive relationships and positive emotions might directly effect the occurrence of growth by creating an emotional and social environment that contributes to positive change processes. Alternatively, they might moderate the direct effect of major life events on growth by fostering positive development that is a direct reaction to the experience.

### Positive emotions

One aspect that distinguishes major life events from daily experiences is the high emotional valence connected to these events. Under stressful situations with positive or negative valence, memory processes are strongly enhanced (Phelps, 2004). These neurological processes increase one's ability to form lasting memories and enhance learning processes (Hu et al., 2007). While positive or negative emotions can facilitate enhanced memory processes (Seng, 2012), research indicates that especially positive emotions are critical for the occurrence of psychological growth (Norlander et al., 2005).

In a longitudinal study, Fredrickson et al. (2003) investigated the influence of positive emotions on trauma-related outcomes of the terrorist attacks on September 11th. They found that participants who reported higher levels of positive emotions before the terrorist attacks were more likely to react with resilience or posttraumatic growth afterwards. Fredrickson explains the relation between positive emotion and post-crises growth with a mechanism based on her *broaden-and-build theory* (Fredrickson, 2004). High prevalence of positive emotions provides the neuronal activation for changes in the brain related to a broadening of thought-action repertoires (Fredrickson, 2004). Positive emotions support coping processes by broadening one's attention, thinking, and behavioral skills (Fredrickson, 2004; Tugade et al., 2004). As a consequence, individuals build long-lasting resources, such as a broader arsenal of coping strategies, deeper relationships, and higher well-being. Hereby, individuals develop critical capabilities to draw from in times of adversity that facilitate posttraumatic growth (Folkman and Moskowitz, 2000; Folkman, 2008). Norlander et al. (2005) found that people who report a high level of positive emotions in daily life are more likely to show posttraumatic growth. In Fredrickson's framework of positive emotions, also psychological peak experiences can lead to increased resilience and psychosocial growth. In this sense, positive emotions are not only a momentarily pleasant experience, but show long-term effects on an individual's cognitive and socio-emotional development.

### Supportive relationships

Another key moderator for human thriving after major life events are supportive relationships (Nez et al., 2010; Schroevers et al., 2010). Social support is one of the critical environmental resources in understanding positive outcomes of life crises (Schaefer and Moos, 1998; Prati and Pietrantoni, 2009). Close relationships may contribute to personal growth by facilitating coping processes and fostering successful adaptation to life crises and challenging events (Prati and Pietrantoni, 2009). Individuals who are surrounded by supportive friends and family members are more likely to integrate the new experience and develop posttraumatic growth (Schroevers et al., 2010). Tedeschi and Calhoun's (1996) primary model of posttraumatic growth included closer social relationships as an outcome variable of PTG. However, in their revised theory, social support also functions as an important predictor of growth after life crises if when it remains stable throughout the coping process (Tedeschi and Calhoun, 2004).

In addition to the importance of supportive relationships for posttraumatic growth, they may also be an important factor for growth after good experiences. Many individuals experience changes after positive events by using a strategy of capitalization and savoring (Bryant, 2003; Bryant and Veroff, 2007). Scales et al. (2011) emphasize that supportive relationships help young people to develop their “sparks in context” (p. 265) and orient them toward thriving. Simultaneously, sharing good experiences improves the relationship with those who participate in them (Gable et al., 2004).

### Meaning making

While positive emotions and good relationships support growth processes, one critical question remains unanswered: How is it possible to integrate a truly threatening or an overwhelming ecstatic experience into one's self? The inability to integrate a new experience into existing mental structures and the necessity to develop a possibility to do so are important elements for the occurrence of growth (Cann et al., 2010; LoSavio et al., 2011; Siegel, 2012). One cognitive process that is likely to build the link between mentally challenging experiences and existing cognitive patterns is meaning making (Park and Ai, 2006). Seligman et al. (2006) emphasize that “A consistent theme throughout meaning making research is that the people who achieve the greatest benefits are those who use meaning to transform the perception of their circumstances from unfortunate to fortunate” (p. 77). Frankl (1992) proposed that finding meaning in a stressful life event is a major facilitator, which helps people to cope more effectively. Meaning making coping is seen as instrumental and one of the core mechanisms of the process underlying posttraumatic growth (Larner and Blow, 2011). “In a general sense, people will have a more positive outcome if they are able to somehow incorporate their traumatic experience into their existing global meaning system” (Larner and Blow, 2011, p. 188). Models of meaning making coping propose that the process of recreating coherent meaning after the violation of existing global meaning can be seen as a main process of posttraumatic growth (Park and Ai, 2006; Larner and Blow, 2011). Also on the positive side, meaning making seems to be one of the major facilitators of postecstatic growth. Roepke's (2013) research emphasized the fact that positive life events are more likely to lead to growth when they evoke a sense of meaning.

The current study used one main mechanism that has been suggested as a key process of meaning making after negative life events: counterfactual thinking. Counterfactual thinking is defined as meaning making by considering alternatives to the past (Kray et al., 2010). Kray et al. (2010) found that counterfactual thinking and creating meaning in life are causally interrelated and that thinking “what, if not…” increases the meaningfulness of major life events. “Reflecting on and mentally undoing moments in which life was profoundly altered is critical for appreciating life transitions.” (p. 108). Wong et al. (2011) state that counterfactual thinking is not just a random quality of cognitive processing; rather, it heightens the meaningfulness of key life events. Most importantly, reflecting on alternative pathways to critical positive or negative turning points produces greater meaning than the direct reflection on the meaning of the event itself (Kray et al., 2010). Fate perceptions (the assumption that an event “was meant to be” or “was meant to happen”) and benefit-finding (the recognition of positive consequences) were identified as independent causal links between counterfactual thinking and the construction of meaning (Kray et al., 2010). Therefore, counterfactual reflection may facilitate an individual's choosing of a point of view of critical life events that identifies the upsides of reality, creates or strengthens a belief in fate, helps to derive more meaning from important experiences and, by this, helps a person to thrive.

### The current research

Which factors facilitate growth after positive as well as negative life events? Existing research on psychological growth processes either considered negative life events as possible facilitators for human thriving (e.g., Joseph and Linley, 2004; Tedeschi and Calhoun, 2004; Park and Ai, 2006) or focused solely on positive life events (Roepke, 2013). The current article is the first scientific study, known to the authors, which systematically examined the connection between posttraumatic and postecstatic growth. It integrates, for the first time, good and bad life events as possible triggers for accelerated positive psychological change.

In the main study (Study 2) we examined whether people who experience psychological growth after traumatic experiences are also more likely to experience growth after positive life events. Furthermore, the study aimed to test the thriver model, which proposes that supportive relationships, positive affect, and meaning making facilitate growth independent of the valence of the event encountered. Since we assumed that the model predicts positive development irrespective of cultural context, it was tested in two different nations, the U.S. and India. There were no specific hypotheses concerning cross-cultural differences beyond the assumption that the model should be generalizable across cultures, while the impact of different events might differ across countries (see Masuda and Holmes, 1967).

In order to test the thriver model and to compare positive and negative events, it was necessary to quantify major life events and their impact in a pre-study (Study 1). We conducted Study 1 in order to retrieve specific ratings for the impact of different life events and use these ratings to weight events reported in Study 2. Also Study 1 was conducted with an Indian and a U.S. sample, since we assumed that there are intercultural differences in the perception of major life events (see Masuda and Holmes, 1967). At the same time we expected that the model is applicable independently from these differences. Both studies had a cross-sectional design to learn more about the associations of posttraumatic and postecstatic growth, before conducting a more extensive longitudinal study in the future.

We propose that it is not the emotional quality of a given event but individual factors which determine the occurrence of growth. The present paper seeks to determine if there are thrivers who show beneficial psychological changes not only after experiencing negative life events but as a result of highly positive experiences as well. It provides a unique contribution to the field by introducing a framework that explains why some people are more likely to grow than others. Our goal is to identify factors that promote positive psychological development that can be influenced by the individual and can be used to help people thrive.

## Study 1

### Materials and methods of study 1

#### Participants

Individuals were recruited online through Amazon Mechanical Turk (MTurk), a web service site provided by Amazon. Users can fill out questionnaires for a modest financial compensation that is directly transferred to their Amazon account. After submitting their informed consent, participants were directed to the online questionnaire. Every person who completed the survey received a $0.50 reimbursement.

The sample consist of *N* = 1154 participants from the U.S. (*n* = 555) and India (*n* = 599) with 50.5% of the American and 54.1% of the Indian participants being female. The mean age was *M* = 32.79 (*SD* = 12.35) in the U.S. sample and *M* = 30.75 (*SD* = 10.27) in the Indian sample. The Indian sample was more highly educated than the American sample with 77.3% of all participants holding a bachelor’s or master's degree, compared to 41.5% in the U.S. sample.

#### Procedures

The first study aimed to quantify and compare the impact of a variety of different major life events. Following the approach of Holmes and Rahe (1967), participants were asked to rate the necessary readjustment to 62 different life events of positive and negative valence. The perceived necessary readjustment to an event was used to estimate the relational impact of different events.

The presented life event list was composed of the original Social Readjustment Rating Scale (SRRQ; Holmes and Rahe, 1967) and the Trauma Assessment for Adults (TAA; Cusack et al., 2004). The list was complemented by positive life events identified in Roepke's (2013) study on postecstatic growth in order to provide a comprehensive measure of trauma and emotional peak experiences. The wording of some original items from the SRRQ had to be modified to correspond to changed life circumstances in the new century (e.g., “Partner beginning or ceasing work outside the home” instead of: “Wife beginning or ceasing work outside the home;” Holmes and Rahe, 1967, p. 214). All participants were provided with the English version of the following original instruction by Holmes and Rahe (1967, p. 213):

*“(A) Social readjustment includes the amount and duration of change in one's accustomed pattern of life resulting from various life events. As defined, social readjustment measures the intensity and length of time necessary to accommodate to a life event, regardless of the desirability of this event*.

*(B) You are asked to rate a series of life events as to their relative degrees of necessary readjustment. In scoring, use all of your experience in arriving at your answer. This means personal experience where it applies as well as what you have learned to be the case for others. Some persons accommodate to change more readily than others; some persons adjust with particular ease or difficulty to only certain events. Therefore, strive to give your opinion of the average degree of readjustment necessary for each event rather than the extreme*.

(C) The mechanics of rating are these: Event 1, Marriage, has been given an arbitrary value of 500. As you complete each of the remaining events think to yourself, “Is this event indicative of more-or less readjustment than marriage?” “Would the readjustment take longer or shorter to accomplish?” If you decide the readjustment is more intense and protracted, then choose a proportionately larger number and place it in the blank directly opposite the event in the column marked “VALUES.” If you decide the event represents less and shorter readjustment than marriage then indicate how much less by placing a proportionately smaller number in the opposite blank. (If an event requires intense readjustment over a short time span, it may approximate in value an event requiring less intense readjustment over a long period of time.) If the event is equal in social readjustment to marriage, record the number 500 opposite the event.”

The original instruction did not include a maximum value to avoid outliers. Therefore, as a robust measure of location and variance, a 5% trimmed mean and 20% Winsorized variance was used to retrieve a rank order list and improve accuracy (Wilcox and Keselman, 2003). To account for intercultural differences, the analysis was conducted separately for the two samples.

To compare life events with positive and negative valence, all events on the list were categorized according to their emotional valence. Ambiguous items, such as “major change in eating habits,” were not included in further analyses. A complete list of the ratings for all events is displayed in Tables 1, 2. The data of a few respondents (*n* = 16) could not be analyzed because they misunderstood the task and provided answers, such as “yes” or “no,” instead of numeric values.

**Table 1:** **Rank order of life events (U.S. sample)**.

**Event**	**Valence**	**Rank**	**TM**	**Winsorized SD**	**TM norm**
Death of a spouse	N	1	885.54	924.06	100
Childhood sexual molestation with pressure or threats	N	2	803.42	702.29	90.73
Childhood sexual molestation (before age 13)	N	3	781.45	621.65	88.25
Forced sexual assault	N	4	692.23	407.82	78.17
Birth of a child	P	5	691.58	372.10	78.10
Birth of the first child	P	6	677.93	284.69	76.56
Death of a close family member	N	7	676.18	362.12	76.36
Forced sexual contact	N	8	668.82	328.35	75.53
Divorce	N	9	609.47	194.16	68.82
Marital separation from mate	N	10	585.13	202.36	66.08
Pregnancy	P	11	567.11	218.81	64.04
Death of a close friend	N	12	566.72	278.33	64.00
Major personal injury or illness	N	13	520.04	230.19	58.73
Attack with a weapon	N	14	517.51	248.79	58.44
Gaining a new family member	P	15	501.92	222.18	56.68
Being fired from work	N	16	501.22	220.12	56.60
Marriage	P	17	500	*	56.46
Serious accident	N	18	486.60	233.00	54.95
Natural disaster	N	19	479.45	243.08	54.14
Foreclosure on a mortgage or loan	N	20	468.76	199.06	52.93
Retirement from work	0	21	459.68	182.26	51.91
Marital reconciliation with mate	P	22	447.18	172.09	50.50
Major change in financial state	0	23	445.29	194.43	50.28
Falling in love	P	24	442.84	233.42	50.01
Living a life dream	P	25	439.43	236.57	49.62
Attack without a weapon	N	26	418.08	224.96	47.21
Major change in the health or behavior of a family member	0	27	406.07	197.60	45.86
Son or daughter leaving home	0	28	392.55	188.97	44.33
Taking on a mortgage greater than $10.000	0	29	376.25	198.75	42.49
Achieving a crucial long-term goal	P	30	373.81	169.44	42.21
Major change in living conditions	0	31	370.40	171.63	41.83
Changing to a different line of work	0	32	368.91	186.87	41.66
Sexual pressure	N	33	365.28	235.07	41.25
Having a spiritual “awakening”	P	34	361.36	244.18	40.81
Major change in the number of arguments with spouse	0	35	353.44	168.36	39.91
Finding a great new job	P	36	346.83	164.74	39.17
Change in residence	0	37	342.62	181.95	38.69
Sexual difficulties	N	38	326.78	189.38	36.90
Witnessed violence	N	39	316.58	203.90	35.75
Major business readjustment	0	40	316.16	190.57	35.70
Taking on a mortgage less than $10.000	0	41	309.06	219.06	34.90
Outstanding personal achievement	P	42	307.21	181.43	34.69
Major change in responsibilities at work	0	43	301.11	122.61	34.00
Partner beginning or ceasing work outside the home	0	44	298.43	146.37	33.70
Major change in working hours or conditions	0	45	289.88	146.55	32.73
Change to a new school	0	46	277.83	160.84	31.37
Meeting an inspiring person	P	47	248.33	165.64	28.04
In-law troubles	N	48	238.59	155.19	26.94
Major change in usual type/amount of recreation	0	49	217.43	134.41	24.55
Major change in social activities	0	50	198.02	120.05	22.36
Major change in sleeping habits	0	51	197.46	119.82	22.30
Major change in eating habits	0	52	194.35	115.77	21.95
Trouble with the boss	N	53	189.74	143.08	21.43
Vacation	P	54	167.99	135.39	18.97
Major change in number of family get-togethers	0	55	159.45	127.08	18.01
Revision of personal habits	0	56	157.72	129.65	17.81
Major change in church activities	0	57	157.62	132.16	17.80
Christmas	0	58	142.38	134.79	16.08
Minor violations of the law	N	59	121.98	108.17	13.77

*TM, 5% trimmed mean; TM norm, normalized 5% trimmed mean, event valence; N, highly negative event; P, positive event; 0, event neutral or unspecified valence*.

**Table 2:** **Rank order of life events (Indian sample)**.

**Event**	**Valence**	**Rank India**	**TM**	**Winsorized SD**	**TM norm**
Marriage	P	1	500	*	100
Death of a spouse	N	2	419.75	303.88	83.95
Birth of the first child	P	3	399.14	266.11	79.83
Pregnancy	P	4	381.73	227.72	76.35
Birth of a child	P	5	379.18	239.81	75.84
Falling in love	P	6	375.34	243.80	75.07
Divorce	N	7	358.32	247.16	71.67
Serious accident	N	8	349.09	247.28	69.82
Meeting an inspiring person	P	9	349.09	192.69	69.82
Finding a great new job	P	10	348.75	196.75	69.75
Sexual pressure	N	11	336.64	229.86	67.33
Childhood sexual molestation with pressure or threats	N	12	335.60	282.09	67.12
Living a life dream	P	13	335.23	213.96	67.05
Marital separation from mate	N	14	334.18	238.58	66.84
Outstanding personal achievement	P	15	332.83	194.51	66.57
Forced sexual assault	N	16	330.33	258.33	66.07
Death of a close family member	N	17	329.94	402.49	65.99
Childhood sexual molestation (before age 13)	N	18	324.39	268.35	64.88
Achieving a crucial long-term goal	P	19	321.00	183.97	64.20
Major personal injury or illness	N	20	320.26	207.06	64.05
Forced sexual contact	N	21	318.86	255.64	63.72
Natural disaster	N	22	316.25	237.64	63.25
Death of a close friend	N	23	314.75	218.78	62.95
Gaining a new family member	P	24	314.30	196.90	62.86
Major change in financial state	0	25	312.67	196.37	62.53
Being fired from work	N	26	309.75	230.01	61.95
Taking on a mortgage greater than $10.000	0	27	306.55	198.50	61.31
Major change in the health or behavior of a family member	0	28	306.26	162.55	61.25
Son or daughter leaving home	0	29	304.86	200.86	60.97
Retirement from work	0	30	303.60	197.23	60.72
Major change in responsibilities at work	0	31	298.45	154.37	59.69
Attack with a weapon	N	32	293.17	218.26	58.63
Major change in living conditions	0	33	293.17	156.38	58.63
Sexual difficulties	N	34	282.59	193.02	56.52
Taking on a mortgage less than $10.000	0	35	282.26	202.64	56.45
Major business readjustment	0	36	280.43	184.40	56.09
Major change in working hours or conditions	0	37	279.39	164.47	55.88
Having a spiritual “awakening”	P	38	278.55	179.53	55.71
Vacation	P	39	277.68	180.33	55.54
Major change in the number of arguments with spouse	0	40	272.82	167.65	54.56
Changing to a different line of work	0	41	270.63	160.30	54.13
Change in residence	0	42	269.69	162.92	53.94
In-law troubles	N	43	269.14	184.43	53.83
Partner beginning or ceasing work outside the home	0	44	268.99	168.63	53.80
Christmas	0	45	263.47	192.01	52.69
Marital reconciliation with mate	P	46	261.99	173.68	52.40
Change to a new school	0	47	249.75	155.05	49.95
Trouble with the boss	N	48	248.32	163.92	49.66
Major change in social activities	0	49	242.67	136.74	48.53
Foreclosure on a mortgage or loan	N	50	238.62	159.45	47.72
Witnessed violence	N	51	234.46	165.79	46.89
Major change in usual type and/or amount of recreation	0	52	232.47	142.17	46.49
Major change in number of family get-togethers	0	53	231.25	157.20	46.25
Attack without a weapon	N	54	229.66	174.45	45.93
Revision of personal habits	0	55	225.15	147.72	45.03
Major change in sleeping habits	0	56	215.61	140.18	43.12
Major change in eating habits	0	57	209.50	140.25	41.90
Major change in church activities	0	58	205.58	146.21	41.12
Minor violations of the law	N	59	201.31	148.20	40.26

*TM, 5% trimmed mean; TM norm, normalized 5% trimmed mean, event valence; N, highly negative event; P, positive event; 0, event neutral or unspecified valence*.

Additionally, to compare the results of the Indian and the U.S. sample, mean values were normalized to a maximum value of 100. In each sample the event with the highest rating was set to a value of 100, while the remaining events were transformed accordingly with the following formula:

(1) $$x_{norm}=(x*100)/\;x_{max}$$

We have chosen a robust mixed model approach to compare the impact of positive and negative events in both countries.

### Results of study 1

Tables 1, 2 show the rank list of the U.S. and the Indian participants.

**Figure 2:**
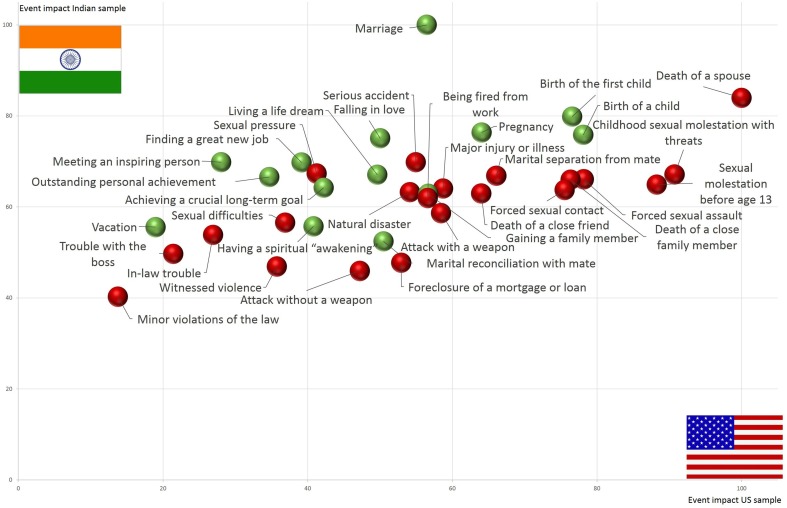
**Results of the event rating for the Indian and U.S. sample**. Green data points represent positive life events, and red data points represent negative experiences.

The U.S. sample showed higher variability, a greater number of outliers, higher trimmed mean values and, for many items, a different rank order than the Indian sample. Outstanding differences occurred for traumatic items linked to sexual abuse and for peak experiences such as falling in love or marriage. While participants of the U.S. sample amplified the impact of sexual molestation, love and marriage played only a minor role in their ranking. The reverse effect could be found in the Indian sample. Both samples showed far-reaching cross-cultural differences in the rating of major life events. A critical finding is the varying importance of negative and positive events in the US and India displayed in Figure 2.

The U.S. sample rated negative events as more impactful (rank, *M* = 20.67, *SD* = 16.05; TM, *M* = 57.51, *SD* = 21.84) than positive experiences (rank, *M* = 24.81, *SD* = 14.37; TM, *M* = 51.12 *SD* = 16.69). Meanwhile, the Indian sample rated negative events as less impactful (rank, *M* = 23.08, *SD* = 15.38; TM, *M* = 64.26, *SD* = 11.50) than positive ones (rank, *M* = 16.90, *SD* = 14.06; TM, *M* = 67.31, *SD* = 8.43). The main and interaction effects of country and valence of events were tested in a robust two-way mixed design with M-estimator and bootstrapping. We found a significant main effects of country ($\hat{\Psi }$ = −127.88, *p* < 0.01), with higher trimmed means values in India, and event valence ($\hat{\Psi }$ = 42.84, *p* < 0.01), with higher values of negative events. However, these main effects should not be interpreted given the highly significant interaction effect of country and event valence ($\hat{\Psi }$ = −125.72, *p* < 0.01). Positive events showed higher impact in the Indian sample than negative ones, while the U.S. sample rated negative events as more impactful.

### Discussion of study 1

Is bad stronger than good? The impact and importance of traumatic experiences has been emphasized and studied extensively, emphasizing the prior role of negative experiences (Baumeister et al., 2001). However, the majority of these findings were based on Western samples or did not investigate positive experiences. Following the approach of Holmes and Rahe (1967), a systematic investigation of the impact of major life events with positive and negative emotional valence was conducted. The life event ranking performed here highlights sociological changes over the last decades as well as intercultural differences. Compared to the results of the original study, some items, such as marriage, lost their importance in the U.S. sample. Additionally, many of the items in the top ranks, such as “sexual molestation” or “birth of a child,” were not even included in the original SRRS list (Holmes and Rahe, 1967). This finding is critical to future research on major life events based on the SRRS, indicating that the original list should be extended and adapted to fit the present times and social situations.

#### Limitations

The original instruction by Holmes and Rahe (1967) did not include a maximum value. Therefore, some participants rated single items with values up to 10,000. Most of these items focused on childhood sexual abuse. Furthermore, the instruction invites participants to rate the impact of these events based on either personal experiences or the experiences of others they know, which might distort the results. Future studies should ask participants to indicate which of the events were rated on personal experiences.

#### Intercultural differences

The social emotional impact of life events differed to a great extent across countries. This may be due to the diverse normativity of certain events, such as the death of a close family member. While in Western countries medical care prevents or treats life threatening illnesses or accidents more effectively, the likelihood of losing a close family member is lower, which might result in higher perceived impact of these events. Moreover, the severity of an event might also be higher or lower because of the consequences specific events have in different countries. Being fired from work might only be a minor impairment in places where a sufficient social security system provides support in times of need. Finally, the perception of the severity of events, such as child abuse, might differ because of media and cultural influences communicating an event as less or more severe.

To account for these intercultural differences in the perception of major life events, results of Study 1 were used to operationalize and compare different events in the two countries in Study 2. The basic assumption was that even though the impact of different events varies to a great extend across countries the facilitating factors of growth are universal.

#### Negativity bias

A particularly intriguing finding is the disparity in the negativity bias (see Rozin and Royzman, 2001). While the results of the U.S. sample support the hypothesis that negative experiences are stronger than positive ones, the Indian sample indicated the reverse effect. These findings raise the question of the universality of the negativity bias and highlight the importance of cross-cultural comparison in basic research.

## Study 2

The main study aimed to test the thriver model and to examine its generalizability and external validity. The model was applied to good and bad experiences in order to investigate its independency from the emotional valence of events.

### Materials and methods of study 2

#### Participants and procedures

Six hundred and three participants from the U.S. (*n* = 342) and India (*n* = 341) enrolled in the study. In this sample 64.3% of the US and 40.2% of the Indian subjects were female. Participants were recruited through the website Amazon Mechanical Turk. Following standard informed consent procedures, they provided responses to a series of questions. The primary dependent variable was the level of reported growth, operationalized by perceived posttraumatic and postecstatic growth. The independent variables were the extent to which participants experienced the potentially facilitating factors of positive emotions, social support, and meaning making in the form of positive counterfactual thinking. Participants provided basic demographic information, such as year of birth, home country, and educational level, as well as basic information about the traumatic and ecstatic events, including what happened and how much time elapsed since the event. After they had submitted this information, participants were redirected to the study survey hosted on the survey software site Qualtrics. Through the Amazon Mturk network, participants received a reimbursement of $0.50. Ethical approval for this study was provided by the institutional review board of the University of Pennsylvania.

**Figure 3:**
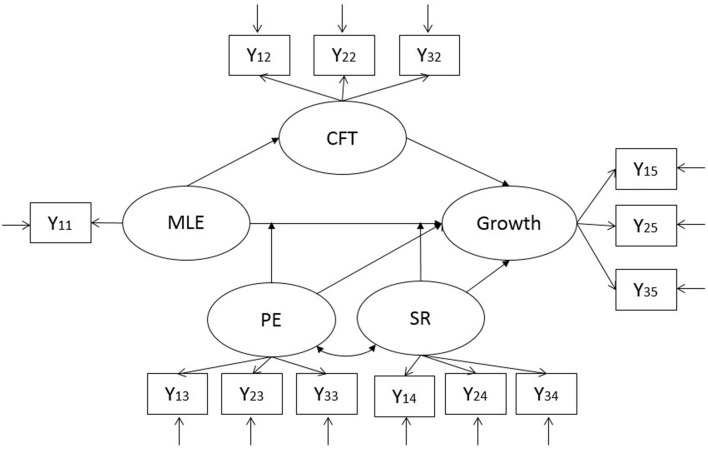
**Applied structural equation model**. This model was applied separately to positive and negative events. Positive events: MLE-P, highest impact of experienced positive event; Growth-P, postecstatic growth; CFT-P, counterfactual thinking about positive experiences; Negative event: MLE-N, highest impact of experienced negative event; CFT-N, counterfactual thinking about negative experiences; Growth-N, reported posttraumatic growth; PE, positive emotions; SR, supportive relationships.

To estimate the model fit of the thriver model, structural equation modeling was applied. The normalized trimmed mean values calculated in Study 1 were used to estimate the impact of the most powerful positive (MLE-P) and negative (MLE-N) events reported by each participant in Study 2. The data from the Indian and U.S. sample in Study 1 were applied for the corresponding group in Study 2. Item parceling was used to create three manifest variables from every scale as indicators of the latent variables of the model (Hall et al., 1999). Five latent variables were included in the SEM testing: impact of major life event (MLE), meaning making in form of counterfactual thinking (CFT), positive emotions (PE), supportive relationships (SR), and reported growth. The resulting structural equation model is displayed in Figure 3. Because there is only one indicator of the latent variable MLE, the factor loading was fixed to 1 and the error variance was fixed to 0. Hence, this variable is equal to its observed indicator.

The factorial structure and the cross-cultural equivalence of the thriver model was tested using multiple group modeling.

### Measures of study 2

#### Negative life events (MLE-N)

A short form of the Trauma Assessment for Adults (TAA; Cusack et al., 2004) has been used to indicate potentially traumatic life events in the past. No particular time frame was given to allow participants to report childhood experiences. The reported events were weighted with the results from Study 1 in the same country. The highest result, indicating the most severe event, was included as indicator for the impact of negative life events (MLE-N). Therefore, the individual score is the trimmed mean of this event from the country to which the individual belongs.

#### Positive life events (MLE-P)

Participants were provided with a list of positive events, such as birth of a child, which had been extracted from Roepke's (2013) study on ecstatic life events. An “other” box has been provided to give subjects the opportunity to choose a life event not included in the list. Comparable to the determination of the MLE-N, participants were not provided with a fixed time frame for the MLE-P. The reported experiences were weighted with the results from Study 1 from the same country. The highest result, indicating the most powerful experience, was included as indicator for the impact of positive life events (MLE-P). Hence, the individual score is the trimmed mean of this event from the country to which the individual belongs.

#### Growth following negative events (Growth-N)

After indicating their most severe experience, participants were asked to estimate how this event changed their lives. The Posttraumatic Growth Inventory (PTGI) measures the perceived positive psychological changes after stressful life events. Participants respond to items such as: “I have a greater appreciation of the value of my own life.” The PTGI has been validated multiple times (Tedeschi and Calhoun, 1996) and shows a good internal consistency with Cronbach's alpha coefficients of α = 0.83–0.91 for its subscales. For the SEM analysis, the subscale “deeper social relationships” was excluded to avoid an overlap with the factor “positive relationships.”

#### Growth following positive events (Growth-P)

After indicating the most positive event they had experienced, participants were provided with the Inventory of Growth after Positive Experiences (IGPE). The IGPE measures the extent to which people feel they have experienced psychological changes because of a positive event in their past (Roepke, 2013). It includes items such as “I have a new role in life.” The inventory has shown good reliability and validity (Roepke, 2013). With *α* = 0.95, the scale had a very good internal consistency.

#### Positive emotions (PE)

The Positive and Negative Affects Schedule (PANAS) measures the extent to which individuals felt negative (e.g., distressed) and positive affects (e.g., interested) during the last month (Watson et al., 1988; Crawford and Henry, 2004). For the present study, only the positive subscale (PANAS-P) was used. The PANAS-P displayed good internal consistency (*α*_US_ = 0.85, *α*_India_ = 0.89).

#### Supportive relationships (SR)

The Multidimensional Scale of Perceived Social Support (MSPSS) measures the extent to which persons feel that they receive support from others, including significant others, family members, and friends. Participants respond to questions such as: “I have a special person who is a real source of comfort to me.” Its reliability and validity are well-established (Zimet et al., 1988). With a Cronbach's alpha coefficient of *α* = 0.92, the scale displayed suitable internal consistency.

#### Counterfactual thinking (CFTI)

The Counterfactual Thinking Inventory (CFTI) asks subjects about their reflection habits when they remember past life events (Mangelsdorf, 2012). The CFTI consists of two subscales for positive (CFTI-P) and negative experiences (CFTI-N). Participants respond on a Likert scale ranging from 1 (*not at all like me*) to 5 (*just like me*) to questions such as “When I think about past life events, I often ask myself where I would be now without these experiences.” Confirmatory factor analyses showed that counterfactual thinking can be distinguished from posttraumatic as well as postecstatic growth. The CFTI-P and CFTI-N showed good internal consistencies (*α* = 0.82 and 0.84).

#### Methods of data analyses

The collected data were analyzed in two steps. First, a correlation analysis was conducted to estimate the relation of posttraumatic and postecstatic growth and to test the interdependence of the constructs, included in the thriver model. Second, the general thriver model was tested separately for positive and negative events with SEM. This approach allowed us to compare its fit for life events with different emotional valence.

### Results of study 2

Table 3 shows the bivariate correlations of the measures that were utilized, while Table 4 displays their descriptive statistics.

**Table 3:** **Intercorrelations of questionnaires used for the validation of the thriver model**.

	**PTGI**	**IGPE**	**MSPSS**	**PAN-P**	**CFTI-P**	**CFTI-N**	**MLE-P**	**MLE-N**
Posttraumatic growth (PTGI)	^*^	0.63^**^	0.41^**^	0.54^**^	0.41^**^	0.38^**^	0.16^**^	0.07
Postecstatic growth (IGPE)	0.67^**^	^*^	0.39^**^	0.54^**^	0.34^**^	0.33^**^	0.18^**^	0.02
Supportive rela-tionships (MSPSS)	0.52^**^	0.48^**^	^*^	0.30^**^	0.26^**^	0.15^**^	0.14^*^	−0.04
Positive emotions (PAN-P)	0.58^**^	0.49^**^	0.46^**^	^*^	0.13^*^	0.10	0.09	−0.04
Counterfactual thinking (CFTI-P)	0.38^**^	0.41^**^	0.26^**^	0.28^**^	^*^	0.57^**^	0.12	0.02
Counterfactual thinking (CFTI-N)	0.39^**^	0.34^**^	0.14^*^	0.26^**^	0.50^**^	^*^	−0.06	0.00
Impact Positive Event (MLE-P)	−0.07	−0.01	−0.10	−0.13	−0.03	−0.02	^*^	0.26
Impact Negative Event (MLE-N)	0.09	0.03	−0.02	−0.04	0.09	0.12^*^	0.12	^*^

The upper triangle shows intercorrelations in the U.S. sample. The lower triangle shows intercorrelations in the Indian sample. PTGI, Posttraumatic Growth Inventory without the subscale social relationships; IGPE, Inventory of Growth after Positive Experiences; MSPSS, Multidimensional Scale of Perceived Social Support; PAN-P, Positive and Negative Affect Scale (positive items); CFTI-P, Counterfactual Thinking Inventory (positive events); CFTI-N, Counterfactual Thinking Inventory (negative events); MLE-P, Impact of the most positive event; MLE-N, Impact of the most negative event;

* p < 0.05;

** *p < 0.01*.

**Table 4:** **Descriptive statistics of the scales used for the thriver model**.

	**PTGI**		**IGPE**		**MSPSS**		**PANAS-P**		**CFTI-N**		**CFTI-P**		**MLE-P**		**MLE-N**	
	***M***	***SD***	***M***	***SD***	***M***	***SD***	***M***	***SD***	***M***	***SD***	***M***	***SD***	***M***	***SD***	***M***	***SD***
US	3.61	1.11	4.04	1.19	5.14	1.29	3.11	0.88	3.22	0.75	3.30	0.84	59.23	17.86	62.78	18.67
India	4.28	0.79	4.76	0.82	5.42	0.96	3.81	0.70	3.34	0.62	3.52	0.60	67.65	16.62	60.50	18.36

*PTGI, Posttraumatic Growth Inventory without the subscale social relationships; IGPE, Inventory of Growth after Positive Experiences; MSPSS, Multidimensional Scale of Perceived Social Support; PANAS-P, Positive and Negative Affect Scale (positive items); CFTI-N, Counterfactual Thinking Inventory (negative life events); CFTI-P, Counterfactual Thinking Inventory (positive life events); MLE-P, Major life event (positive); MLE-N, Major life event (negative)*.

Because the number of positive and negative events were also assessed in Study 2, we checked whether the number of events is important for growth. While the number of negative events showed no significant relations to posttraumatic (*r* = −0.01, *p* = 0.79) or postecstatic growth (*r* = −0.04, *p* = 0.29), the number of positive events was positively associated with postecstatic (*r* = 0.10, *p* = 0.01) as well as posttraumatic growth (*r* = 0.17, *p* < 0.01).

One of the key questions underlying the present research focuses on the relation between posttraumatic and postecstatic growth, which has not been studied before. The two constructs showed high intercorrelations between *r* = 0.63 (Indian sample) and *r* = 0.67 (U.S. sample). This finding supports the hypothesis that posttraumatic and postecstatic growth are highly interrelated and possibly cognate processes.

Growth after positive and negative events was significantly correlated with all facilitating variabes included in the thriver model. At the same time, the impact of the most severe negative event showed no significant correlation with posttraumatic growth. This result remains stable, also when the most severe traumatizing events were excluded from the sample. Most participants (87.7%) reported that they experienced at least one traumatic event included in the TAA (Cusack et al., 2004). An interesting finding is that participants who experienced a more impactful positive event also reported more posttraumatic growth, but only in the U.S. sample. In general, however, the degree of growth after positive and negative events depends only marginally on the impact of the event. This finding can be taken as hint that a person's capacity for growth seem to be more important than the impact of the event itself. We conclude, therefore, that the experience of a major life event is a nessicity to ignite multi-dimensional growth but the degree of growth seems to depend more on the facilitating factors than on the event itself.

The descriptive statistics, displayed in Table 4, revealed consistent cross-cultural differences [MANOVA: *F*_(8, 571)_ = 20.47, *p* < 0.001; Pillai-Trace for difference between the two nations: η^2^ = 0.22].

The U.S. sample showed lower mean values than the Indian sample for all scales except the one assessing the impact of negative events (MLE-N). In sum, we found strong correlations between PTG and PEG as well as the faciliatating factors of the thriver model. These findings support our hypotheses.

#### Structural equation modeling

Multiple group structural equation modeling (SEM) using the computer program Mplus (Muthén and Muthén, 1998–2012) was applied to analyze the thriver model for positive and negative experiences. First, measurement equivalence was tested across countries (Byrne, 2008). Second, the fit of the thriver model for positive and negative events was tested for the Indian and the U.S. sample in a multigroup analysis.

Mplus does not provide model fit coefficients for models with latent interaction variables. Therefore, in the first step, we tested the model in Figure 2 without moderating effects in order to see whether the general model structure fits the data (estimator: MLR). Next, the moderation hypotheses were tested (estimator: MLR). If the moderation effects were not significant, the moderation effects were excluded again to simplify the models. The path coefficients of all effects, including moderations, are reported below.

#### Structural equivalence

It was possible to establish configural equivalence for negative [χ^2^_(120)_ = 141.86, *p* = 0.08, CFI = 0.99; TLI = 0.99; RMSEA = 0.024] and positive events [χ^2^_(120)_ = 160.09, *p* = 0.01, CFI = 0.99; TLI = 0.99; RMSEA = 0.037]. These findings indicate a good fit of the thriver model in the Indian and the U.S. sample.

The results for metric equivalence showed acceptable model fit results [negative events: χ^2^_(132)_ = 202.80, *p* < 0.01, CFI = 0.98; TLI = 0.98; RMSEA = 0.042; positive events: χ^2^_(132)_ = 209.97, *p* < 0.01, CFI = 0.98; TLI = 0.98; RMSEA = 0.05]. However, the chi-square (χ^2^) difference tests showed that the assumption of metric equivalence has to be rejected for positive (χ^2^_diff_ = 45.23, df_diff_ = 12, *p* < 0.01) and negative events (χ^2^_diff_ = 65.39, df_diff_ = 12, *p* < 0.01). Therefore, more restrictive models of measurement equivalence were not tested. The estimated intercept and factor loadings in Tables A1, A2 in Appendix show that there are only very small differences between the two countries, so that approximate measurement invariance is given. Therefore, it is meaningful to compare the estimated parameters between the two countries.

#### The thriver model

**Figure 4:**
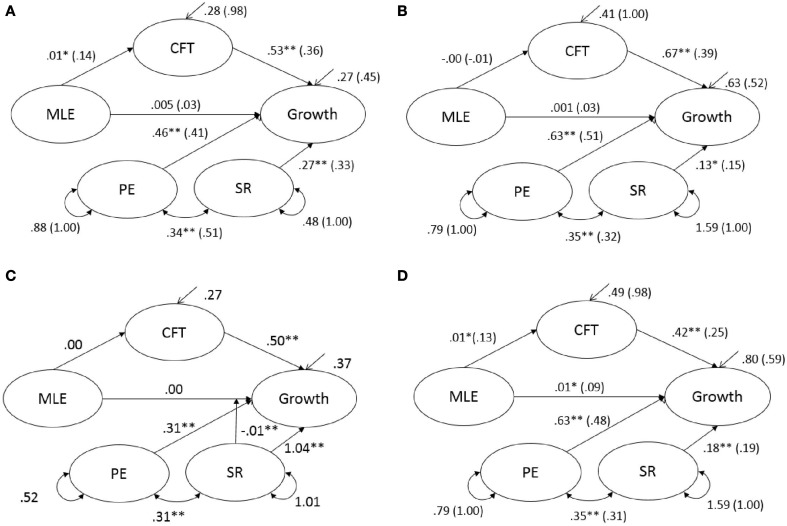
**Estimated Q13 model parameters of the thriver model for positive and negative events in the U.S. and India**. **(A–D)** Structural model for negative events in India; unstandardized (Standardized) results. ^*^p < 0.05; ^**^p < 0.01.

The thriver model was tested for positive and negative events in the U.S. and in India. All direct path coefficients of the facilitating factors were positive, relatively large, and significantly different from 0. Analyses of the full model for negative events, including interaction effects, showed highly significant direct effects but no significant interaction effect of the moderators positive emotions (β_US_ = −0.003, *p* = 0.48; β_India_ = −0.01, *p* = 0.19) or social relationships and MLE (β_US_ = −0.002, *p* = 0.38; β_India_ = −0.004, *p* = 0.32). Also, for positive events the interaction effect of positive emotions (β_US_ = −0.006, *p* = 0.13; β_India_ = 0.001, *p* = 0.07) was not significant, while the interaction of social relationships and MLE was only significant in one condition (β_US_ = 0.001, *p* = 0.73; β_India_ = −0.01, *p* < 0.01). The value of the significant interaction effect is small because of the large range of the variable MLE. This interaction effect indicates that for positive events with increasing impact in India, more social support does not necessarily lead to more postecstatic growth. The estimated model parameters are presented in Figures 4A–D.

In all four conditions, the direct effect of major life events on growth was small or not significant, indicating that other supporting factors facilitate growth in addition to the impact of an event. In the U.S. sample, positive emotions had the strongest relation to growth, followed by meaning making, while relationships played a minor role. In the Indian sample, all three facilitating variables were about equally important in predicting growth, while positive emotions were especially important concerning negative events. Meaning making through counterfactual thinking was a critical predictor for growth in both populations after bad as well as good experiences. While counterfactual thinking is based on the reflection of major life events, it showed only small or no significant relation to the impact of the most severe event. Overall, the thriver model showed a good model fit in all four analyses.

#### Discussion of study 2

Are there people who are more likely to grow than others? Study 2 aimed to answer this question and to test a novel model that unifies contributing factors to growth after good and bad experiences. The results highlight that the experience of posttraumatic and postecstatic growth are highly interrelated. Participants who reported positive changes after trauma also experienced positive development after peak experiences and could be identified as *thrivers*. This finding was generalizable across the investigated countries, and exemplifies the overlap of the two constructs. Future research should aim to disentangle and further elaborate on the relationship of growth after positive and negative events.

#### The thriver model

The current cross-sectional results support the main assumptions of the thriver model. All three factors included in the model as facilitators of positive development are related to growth processes after highly positive as well as traumatic experiences. While the extent to which the different factors explain the occurrence of growth slightly differs across countries and events, the general model showed a good fit in all four conditions. Supportive relationships and positive emotions could not be identified as moderators, but had a highly significant direct effect on growth. Therefore, in the future, the model should be tested in longitudinal designs to verify the directionality of these relations.

#### Major life events

Another intriguing finding of the second study concerns the missing link between events and growth. Especially after negative experiences, the mere occurrence of a given event does not predict positive development. It seems that major life events result in a psychological disruption, which only leads to growth when they are accompanied by certain supporting factors. This finding emphasizes the critical influence of facilitating factors contributing to positive outcomes.

The findings concerning relations between the number of positive and negative events and growth questions once more the prior role of negative experiences. They indicate that experiencing numerous negative events often does not lead to more growth. At the same time, even though more positive experiences might also be a consequence of growth, the positive relation between growth and positive life events might be based on the beneficial effects of positive experiences. Possibly, for long-term developmental outcomes, positive experiences are more critical than negative ones, since they facilitate growth and buffer the impact of adversarial events (Folkman and Moskowitz, 2000; Folkman, 2008). Future research should investigate the causal direction of this effect.

Significant relationships between impact of an event and the occurrence of counterfactual thinking or growth could only be found for positive events in the U.S., and negative events in India. It seems that meaning making is a process which may be facilitated by means other than the character of the event itself, such as the necessity to reappraise a situation (Park and Ai, 2006). Likewise, does the mere fact that a certain event happened not automatically lead to positive development without adaptive processing of the experience.

The good model fit in both countries and the fact that the path coefficients do not differ a great deal between the nations lead to the conclusion that the thriver model is generalizable across both countries and, therefore, at least to some degree independent of cultural context.

## General discussion

Major life events are an integral component of every person's life. They often not only alter one's biography, but the person as a whole. The thriver model is a new perspective on positive human development. It provides a framework to think about development as not only a continuous learning process or rapid changes, but as an interaction process between external influences, internal processing mechanisms, and psychological resources.

The current studies were the first systematic investigation of the relationship between posttraumatic and postecstatic growth. The findings suggest that both concepts are highly interrelated and that there are personal factors that drive positive changes independent of an event's valence. This raises the question of whether the good or bad character of an event is critical for the positive outcomes that may occur. Possibly, the terminologies of posttraumatic and postecstatic growth are misleading, since they limit the phenomenon of growth to the specific emotional valence of an experience. Future research should aim to disentangle the mechanisms and components of positive changes after good and bad experiences to determine which are specific to the character of an event and which are universal. Possibly, there are beneficial outcomes which are distinct for positive and negative life events. One of the main goals of future research should also be to test the model in longitudinal settings and hereby clarify how the different components of the model unfold and interact.

### The thriver model

For this study, we developed a general model to help explain the occurrence of personal growth. The thriver model is based on the assumption of within-person factors that are critical for positive development after positive and negative life events. These factors were merged into a theoretical model, which was applied not only to varying life events but also in two different cultures. The results support the hypotheses that PTG and PEG are not only highly interrelated, they might also be facilitated by parallel psychological factors.

The thriver model aims to explain, predict, and help to enable positive changes after major life events. It complements other taxonomies of well-being, including Seligman's PERMA (2011) theory and Ryff's concept of psychological well-being (Ryff and Keyes, 1995), by adding a process perspective on the question of a well-lived life. The current studies were a first step to test the *thriver* model in a cross-cultural comparison approach. The key components of meaning making, supportive relationships, and positive emotions showed strong relations with self-reported growth in the U.S. and in the Indian sample. These results are supported by earlier research on turning points, which identified meaning making in times of life-altering experiences as a critical facilitator for psychological well-being (Tavernier and Willoughby, 2012). The results are also concurrent with narrative attachment research which emphasizes the prior role of close relationships and high effectance motivation in adults (Sabir, 2014). Finally, the outcomes are in accordance with Fredrickson's (2004) work on the facilitating role of positive emotions for personal development. In sum, *thrivers* might be described as persons with a well-developed ability to create meaning from their experiences, a secure attachment status, and a high positivity ratio.

The model was generalizable across positive and negative life events in different countries and has shown its broad applicability. The direct effects of all three key variables where highly significant. In most cases, it was not possible to clearly identify them as mediators or moderators for the occurrence of growth. An explanation for this finding might lie in the relationship between perceived distress and the occurrence of growth. In our study, we used the impact of the life event as a proxy to operationalize life events as a continuous variable. While some research finds that more distress of events (which would result in higher impact) is related to more PTG (Cordova et al., 2001), others indicate that there might be a curvilinear relationship between distress and PTG (Lechner et al., 2003). This might also explain why the direct effect of major life events on growth was either small or not significant. An alternative explanation would be that major life events are a necessary but not sufficient prerequisite for growth, which only occurs when they are accompanied by specific psychological resources. Future research should therefore aim to disentangle the direction in which the different factors interact to enable growth processes in a longitudinal design.

### Limitations and outlook

#### Design and time scale

The current studies aimed to test the thriver model and to determine if longitudinal and thus more expensive future projects are justifiable. Therefore, we conducted both studies in a cross-sectional design. This approach led to shortcomings, which should be addressed in future research. The thriver model is a process model, which ultimately aims to inform interventions designed to help people grow. At the same time, it is not possible to investigate a process model exhaustively and to verify causations between observed variables without longitudinal observations. In addition, all variables were measured at the present time and did not retrospectively refer to the time of the life event. This approach was taken because we did not assume that participants would be able to recall retrospectively their meaning making mindset, positive emotions, and social relations when the life event happened. This cross-sectional approach leaves the question unanswered if a high level of positive emotions, social support, and meaning making are a consequence of or prerequisite for the occurrence of growth. It might also be possible that having a high level of all three variables at the measurement time influences how participants evaluate the life events in their past. Subsequent studies should, therefore, have different measurement time points that include pre- and post-event data as well as a follow-up measures for single events in order to disentangle the underlying mechanisms. Meanwhile, the present results indicate that it will be of value to conduct longitudinal research on these mechanisms.

#### Perceived growth

The current studies were based on measures of perceived growth. Increasingly, researchers expressed their doubts if perceived posttraumatic growth actually mirrors genuine growth (Frazier et al., 2009). Park and Helgeson (2006) refer to this problem as a veridicality issue, emphasizing that some reports of growth may represent cognitive distortions or illusions, rather than genuine growth. One provided alternative explanation is that self-reported growth experiences are motivated illusions with the inherent goal to alleviate distress through self-enhancement (McFarland and Alvaro, 2000). This approach assumes that posttraumatic growth is primarily a cognitive coping strategy, reducing the negative impact of stressful life events. However, while this explanation may be applicable to negative life events, it does not explain why people report growth after positive experiences as well. To disentangle genuine growth from perceived growth as a coping strategy, future research should measure the different domains of growth with scales that are not related to the event itself in a pre- and post-test design.

#### Non-exhaustive approach

The thriver model unifies three key factors, which showed high explanatory power for positive changes after threatening and highly positive events. However, there may be other contributing factors, which might also be considered in future research, such as an individual's personal initiative to thrive (i.e., personal growth initiative; Robitschek, 1998) or effectance motivation (Sabir, 2014).

In sum, the current studies can be considered a critical first step to study the connections between posttraumatic and postecstatic growth in order to disentangle the complex mechanisms underlying positive development across the life span.

### Conclusions

The thriver model unites key components that contribute to positive development independent of the life path encountered. It explains growth processes that occur after turning points in life with positive or negative valence. Drawing from beneficial processes following life's best and worst moments, it identifies three factors that might not only enable positive changes after major life events, but can also help people to make the most of the experiences they have had in their life. Taking both good and bad experiences into consideration as possible facilitators of growth may broaden our understanding of the origin of positive human development. Following the saying: “We can't change the cards we are dealt, just how we play the hand,” it may be the basis for a new line of interventions, enabling people to benefit from whatever they may encounter in life.

### Conflict of interest statement

The authors declare that the research was conducted in the absence of any commercial or financial relationships that could be construed as a potential conflict of interest.

## Appendix

**Table A1:** **Factor loadings and standard error of parcels used for the thriver model (negative events)**.

**Negative life events**		**India**			**USA**		
		**Estimate**	**S.E**.	**Est./S.E**.	**Estimate**	**S.E**.	**Est./S.E**.
Growth (PTG)	PTGI_1	1.00	0	999.00	1.00	0	999.00
	PTGI_2	0.91	0.05	18.41	0.91	0.04	24.64
	PTGI_3	0.97	0.05	19.01	0.98	0.04	26.12
SR	MSPSS_1	1.00	0.00	999.00	1.00	0.00	999.00
	MSPSS_2	1.00	0.05	20.79	1.00	0.03	33.82
	MSPSS_3	0.94	0.05	18.82	0.98	0.03	35.50
PE	PANAS_P1	1.00	0	999.00	1.00	0.00	999.00
	PANAS_P2	0.92	0.06	15.53	0.94	0.04	21.65
	PANAS_P3	0.94	0.05	17.32	0.94	0.04	23.89
CFT-N	CFTI-N1	1.00	0.00	999.00	1.00	0.00	999.00
	CFTI-N2	0.93	0.14	6.77	1.01	0.14	7.92
	CFTI-N3	0.97	0.16	6.01	0.85	0.14	6.26

*Growth, Posttraumatic growth (PTGI); SR, Supportive relationships (MSPSS); PE, Positive emotions (PANAS-P); CFT-N, Counterfactual thinking (CFTI, negative events)*.

**Table A2:** **Factor loadings and standard error of parcels used for the thriver model (positive events)**.

**Positive events**		**India**			**USA**		
		**Estimate**	**S.E**.	**Est./S.E**.	**Estimate**	**S.E**.	**Est./S.E**.
Growth (PEG)	IGPE_1	1.00	0	999.00	1.00	0.00	999.00
	IGPE_2	1.02	0.05	22.69	0.98	0.02	49.80
	IGPE_3	1.01	0.04	25.38	0.97	0.02	49.70
SR	MSPSS_1	1.00	0.00	999.00	1.00	0.00	999.00
	MSPSS_2	0.99	0.06	16.39	1.03	0.03	34.37
	MSPSS_3	0.91	0.06	15.99	0.99	0.03	35.47
PE	PANAS_P1	1.00	0.00	999.00	1.00	0.00	999.00
	PANAS_P2	1.02	0.05	22.69	0.94	0.04	22.04
	PANAS_P3	1.01	0.04	25.38	0.94	0.04	23.93
CFT-P	CFTI-P1	1.00	0.00	999.00	1.00	0.00	999.00
	CFTI-P2	1.14	0.15	7.63	1.27	0.11	11.32
	CFTI-P3	1.05	0.14	7.63	1.00	0.09	11.08

*Growth, Postecstatic growth (IGPE); SR, Supportive relationships (MSPSS); PE, Positive emotions (PANAS-P); CFT-P, Counterfactual thinking (CFTI, positive events)*.
